# Targeted Therapy in Advanced and Metastatic Non-Small Cell Lung Cancer. An Update on Treatment of the Most Important Actionable Oncogenic Driver Alterations

**DOI:** 10.3390/cancers13040804

**Published:** 2021-02-15

**Authors:** David König, Spasenija Savic Prince, Sacha I. Rothschild

**Affiliations:** 1 Department of Medical Oncology, University Hospital Basel, 4031 Basel, Switzerland; david.koenig@usb.ch; 2 Comprehensive Cancer Center, University Hospital Basel, 4031 Basel, Switzerland; spasenija.savicprince@usb.ch; 3 Pathology, Institute of Medical Genetics and Pathology, University Hospital Basel, 4031 Basel, Switzerland

**Keywords:** NSCLC, oncogenic alterations, targeted therapy, KRAS mutations, KRAS G12C inhibitors, EGFR mutation, EGFR exon 20 insertions, ALK gene rearrangements, BRAF mutations, HER2 alterations, ROS1 gene rearrangements, RET gene rearrangements, MET alterations, NTRK gene fusions

## Abstract

**Simple Summary:**

The treatment of advanced and metastatic non-small cell lung cancer (NSCLC) has changed dramatically in recent years due to advanced molecular diagnostics and the recognition of targetable oncogenic driver alterations. This has led to the development of very effective new targeted agents, and thus to a relevant progress in the treatment of oncogene-addicted NSCLC. While the treatment of EGFR-mutated and ALK-rearranged NSCLC is well-established, new targeted therapy options have emerged for other oncogenic alterations. In this comprehensive review article, we discuss the major molecular alterations in NSCLC and the corresponding therapeutic options.

**Abstract:**

Due to groundbreaking developments and continuous progress, the treatment of advanced and metastatic non-small cell lung cancer (NSCLC) has become an exciting, but increasingly challenging task. This applies, in particular, to the subgroup of NSCLC with oncogenic driver alterations. While the treatment of epidermal growth factor receptor (EGFR)-mutated and anaplastic lymphoma kinase (ALK)-rearranged NSCLC with various tyrosine kinase inhibitors (TKIs) is well-established, new targets have been identified in the last few years and new TKIs introduced in clinical practice. Even for KRAS mutations, considered for a long time as an “un-targetable” alteration, promising new drugs are emerging. The detection and in-depth molecular analysis of resistance mechanisms has further fueled the development of new therapeutic strategies. The objective of this review is to give a comprehensive overview on the current landscape of targetable oncogenic alterations in NSCLC.

## 1. Introduction

Lung cancer is by far the leading cause of cancer-related mortality in both male and female worldwide, accounting for nearly 20% of all cancer deaths in Europe [1]. Most represent non-small cell lung cancer (NSCLC, 85%) with adenocarcinoma (AC), squamous cell carcinoma (SCC) and large cell neuroendocrine carcinoma (LCNEC) being the most common morphological subtypes [2]. This purely morphological classification has been challenged in the past decades, as particularly AC can be further subdivided into distinct molecular subtypes. These molecular subtypes are defined by a single oncogenic driver alteration, comprising gene mutations, rearrangements, and amplifications [2]. There is a significant variability in the incidence of targetable oncogenic driver alterations, which mainly occur in AC, are very rare in SCC and are overall more prevalent in Asian populations [3,4]. Molecular subtyping has become highly relevant, as genotype-driven therapy (“targeted therapy”) is nowadays standard of care for a significant subgroup of patients with advanced and metastatic NSCLC (stage IV), which has led to unprecedented outcome improvements. Historically, the estimated median overall survival for patients with stage IV NSCLC was 6 to 10 months [5]. The discovery of activating epidermal growth factor receptor (EGFR) mutations as the first targetable and predictive oncogenic driver alteration in lung cancer, has profoundly changed the diagnostic work-up and the therapeutic landscape of lung AC [6,7]. Treatment of EGFR-mutated NSCLC with EGFR-tyrosine kinase inhibitors (TKIs) is a model for biomarker-driven therapy and highlights the importance of establishing a predictive molecular biomarker for personalized therapy in the treatment of solid tumors [8]. Since the discovery of EGFR mutations in 2004, several other oncogenic driver alterations have been identified. This led to the development of very effective new targeted agents, and thus to a relevant progress in the treatment of oncogene-addicted NSCLC. The decline in mortality from NSCLC observed in the last years has been attributed mostly to improved survival due to novel treatment options such as effective targeted therapy [9,10]. 

Although there are a variety of molecular NSCLC subtypes, oncogene-addicted NSCLC share some typical hallmarks and similarities:

- Generally, oncogenic driver alterations are mutually exclusive, which means that the presence of one oncogenic driver event excludes the presence of another active oncogenic driver. This concept is based on the model that a single, early genomic driver event leads to a state of oncogene addiction, which drives tumorigenesis and tumor progression. Therefore, the respective proteins are ideal targets for anticancer drugs.
- Treatment outcome with targeted therapies is superior to conventional chemotherapy. Randomized phase III trials in different settings have shown significantly improved progression-free survival (PFS) and overall survival (OS).
- Generally, TKIs, selected by a predictive targetable oncogenic driver alteration, yield a high objective response rate (ORR). The magnitude varies, but usually the ORR ranges between 50% and 90% as will be described in the following chapters.
- Responses to TKIs occur timely after onset of therapy, usually within 4–6 weeks.
- Emergence of secondary resistance to targeted therapy is inevitable and usually occurs within 12–24 months. Molecular re-analysis at time of progression for characterization of acquired resistance mechanisms has led to new therapeutic options for various molecular subtypes.

The present review provides an overview on targeted therapy options in advanced and metastatic NSCLC with oncogenic driver alterations.

## 2. Predictive Biomarker Testing for Targeted Treatment

With significant progress in treatment options for patients with advanced, inoperable stage NSCLC, predictive biomarkers, including the morphological subtype, the PD-L1 status and the results of targetable oncogenic driver alterations, are a prerequisite for the decision on the best possible therapy as they are directly linked to the choice of a specific treatment [11,12]. Though current guidelines focus on testing of genes for approved drugs like EGFR, ALK, ROS1 and BRAF, they acknowledge the rapidly changing treatment landscape and suggest to perform a broader molecular profiling including genes like MET, RET, NTRK, KRAS and HER2 to allow for enrolment in clinical trials and for access to off label use of drugs, if an access program is available [11,12]. The Federal Drug Agency (FDA) has just recently approved drugs targeting NSCLC with MET exon 14 skipping alterations and RET and NTRK 1/2/3 rearrangements, respectively. 

Guidelines recommend that all patients with advanced or metastatic non-squamous cell carcinoma of the lung (AC, NSCLC not otherwise specified, large cell carcinoma, adenosquamous carcinoma) should be tested for targetable oncogenic driver alterations [11,12,13]. Routine molecular testing is not recommended in pure SCC, except in selected patients with never-/light-smoking history or young age and diagnosed by small samples. In this setting there is the possibility of an underlying adenosquamous carcinoma, were the glandular component might not have been sampled [11,12,13]. Adenosquamous carcinomas are rare tumors (0.4–4% of all lung cancers), which can harbor the same targetable oncogenic driver alterations as pure AC [2]. Primary tumors or metastatic lesions are equally well suited for predictive testing as oncogenic driver alterations represent key clonal events [13,14].

Typically, advanced stage lung cancer patients are diagnosed by small biopsies and cytology specimens and generally provide enough tumor material for predictive testing. Of note, cytology specimens as a source for predictive molecular biomarker testing is important, as up to 40% of all lung cancers are diagnosed by cytology alone. Conventional cytology preparations, due to the lack of formalin fixation, yield high-quality DNA and are very well suited for sequencing analyses [15]. Current guidelines acknowledge that any cytology preparation, conventional or cell blocks, can be used for predictive molecular testing [13]. Several pre-analytical factors can affect the performance of nucleic acid sequencing [16]. For example, bone biopsies from metastatic lesions should ideally undergo EDTA-based decalcification, which preserves tumor cells for molecular analyses. In contrast, strong acid-based decalcification protocols are not suitable for PCR-based molecular testing. 

To detect the increasing number of treatment relevant molecular targets, frontline simultaneous targeted DNA- and RNA-based next generation sequencing (NGS) is most straightforward as a large panel of relevant genes can be tested for mutations, fusions and amplifications in a timely manner [17]. Total nucleic acid isolation in a single procedure from the same tumor starting material is most tissue sparing. But still, due to the vulnerability of RNA, a significant portion of small NSCLC samples are not suited for rearrangement testing by RNA-based NGS [18,19]. DNA is more stable than RNA and DNA-based NGS panels for comprehensive molecular testing are available allowing for simultaneous assessment of gene mutations, structural rearrangements at the DNA-level and copy number alterations [19]. However, the sensitivity of DNA-based NGS for gene fusion detection is inferior to RNA-based assays, as fusion breakpoints involving long intronic regions or containing repetitive regions cannot be fully covered [20,21,22]. For example, in a cohort of 232 AC an FDA-approved DNA-based NGS assay missed 27 targetable gene fusions (11.5%) and 6 MET exon 14 skipping mutations (2.5%), which were retrospectively uncovered by targeted RNA sequencing using Archer technology [20]. There are several other methods at disposal for the detection of gene rearrangements [23]. For example, single gene rearrangements can be detected on the protein level by IHC or on the DNA level by fluorescence in-situ hybridization (FISH). Both of these methods are applicable to samples with a low number of tumor cells, not suited for RNA-based NGS. Additionally, both of these methods are applicable to histology and cytology specimens, provided that the tests have been validated and continuous quality assurance is performed. 

IHC for ALK and ROS1 is widely used and has been introduced more recently for NTRK fusions and can significantly simplify large-scale screening for these rare gene rearrangements [13,23,24]. 

ALK IHC, using 5A4 or D5F3 antibody clones, is an equivalent alternative to FISH, which used to be the gold standard for ALK testing [13]. In NSCLC with unequivocal ALK staining, ALK targeted therapy can be initiated, without confirmation of the ALK rearrangement by a molecular method. For ROS1 IHC the sensitivity and specificity of the D4D6 clone ranges from 94–100% and 87–100%, respectively, though there are still significantly fewer studies on the performance of ROS1 IHC as compared to ALK [13]. Therefore, ROS1 IHC may be used as a screening test, but positive ROS1 IHC results should always be confirmed by a molecular method [13]. NTRK includes three genes, NTRK 1, 2, and 3, which encode the transmembrane receptor tyrosine kinases TRK A, B, and C, respectively, all of which can be detected by pan-TRK antibodies [25]. Using the pan-TRK antibody clone EPR17341 the largest study so far showed a high sensitivity for detecting NTRK 1 and 2 fusions (96% and 100%, respectively) and a lower sensitivity for NTRK 3 (79%) [26]. Additionally, the performance of pan-TRK IHC differed between various tumor types. For lung cancer the sensitivity and the specificity were 88% and 100%, respectively [26]. Pan-TRK IHC can be used for screening NSCLC, were NTRK rearrangements are very rare (0.2%), with confirmation of any positive staining (even if only present in 1% of tumor cells) by a molecular method, preferably by RNA-based NGS [24,26]. FISH is not a practical screening method for NTRK rearrangements, as it requires separate hybridizations with three different break-apart probes to cover all NTRK genes (1/2/3). In NSCLC wild type for other driver alterations (EGFR, KRAS, ALK, and ROS1), molecular confirmation of a negative pan-TRK IHC staining might be considered, especially in young patients, as pan-TRK IHC has a lower sensitivity for NTRK 3 rearrangements.

There is no established antibody for the detection of RET rearrangements by IHC and RET testing is performed by break-apart FISH, RNA-based NGS or reverse-transcriptase PCR [27]. 

Another recently introduced predictive marker, as mentioned above, are MET exon 14 alterations. Genomic alterations in MET exon 14 and its flanking introns cause MET exon 14 skipping during pre-mRNA splicing with loss of the juxtamembrane domain containing the Y1003 residue that serves as a binding site for the E3 ubiquitin ligase CBL. This results in impaired MET degradation with increased half-life of the active MET receptor and extended downstream MET signaling [28]. There are a large variety of MET genomic alterations that lead to exon 14 skipping. These can be detected on DNA level analyzing exon 14 and its flanking regions by NGS or Sanger sequencing, or on RNA level directly detecting the exon 14 skipping by NGS [29,30]. Of note, it has been shown that due to inadequate primer design, DNA-based NGS using amplicon-mediated target enrichment can miss up to 60% of MET exon 14 alterations, detected by an RNA-based assay [31,32]. This low sensitivity can be caused due to variants found outside of the amplified area(s) or due to the presence of deletions, which prevent proper primer binding [31]. Though MET exon 14 skipping mutations are supposed to lead to increased MET protein presence, there is growing evidence that there is a poor association between MET expression and MET exon 14 alterations. This suggests that MET IHC is insufficient for pre-selection of NSCLC for further MET exon 14 testing [33,34]. 

Another mechanism of oncogenic MET activation is MET amplification [35]. Using FISH there is no clear consensus about the cutoff to define MET amplification, however higher MET gene copy to chromosome 7 centromere (CEP7) ratios of ≥4 seem to better predict responses to treatment with MET inhibitors [36]. The optimal molecular test and cutoff to define MET amplification are still to be defined [30]

In the setting of acquired resistance under TKI treatment, resistance mechanisms develop in a subclonal fashion and might vary between different tumor sites [37]. Liquid biopsy of blood samples with analysis of circulating tumor DNA (ctDNA) in plasma allows to capture this spatial tumor heterogeneity and to detect genetic alterations, which might be missed by random biopsy of one tumor site [38]. Additionally, sequential re-analysis can easily be performed, allowing for monitoring of treatment response and detection of resistant subclones under TKI-treatment. Liquid biopsy has entered routine clinical practice for the detection of T790M EGFR mutations, the most common secondary resistance mutation to first- and second-generation EGFR-TKIs, which triggers adjustment of therapy to osimertinib [13,39]. With the complexity of possible molecular resistance mechanisms across different oncogenic driver alterations and different TKIs, which in general include on target secondary mutations and off target pathway activations, cfDNA testing methodologies have moved from single gene assays to highly sensitive NGS panels [40]. Also for patients, who have inadequate tissue DNA available for initial molecular testing, liquid biopsy offers a non-invasive approach for molecular profiling [13,41,42]. Importantly, the detection of a targetable mutation in ctDNA is sufficient to initiate targeted treatment [43]. As the sensitivity of liquid biopsy is still lower than in tissue samples and detection of gene fusions and amplifications is challenging, a negative liquid biopsy cannot exclude a false negative result and requires molecular testing of a tissue sample [43,44]. In the setting of secondary resistance, a progressing tumor lesion should be sampled. Again, molecular testing should include detection of mutations, rearrangements and amplifications and a NGS panel, which covers the gene and drug-specific molecular resistance alterations has to be chosen. Detection of small cell transformation should not be delayed, and timely tissue sampling performed, whenever there is clinical or radiological suspicion and irrespective of liquid biopsy results. Also, the possibility of false positive liquid biopsy results caused by clonal hematopoiesis, which can involve genes mutated in lung cancer (like TP53 and KRAS), or a mutation from a secondary independent malignancy should be kept in mind [43,45]. 

In summery simultaneous frontline DNA- and RNA-based NGS for mutation, rearrangement and amplification testing is most straightforward. However, testing algorithms might need to be flexibly adapted and methods combined to adjust to the needs of the available tumor material, the available testing methods, the local economic situation and to access to drugs. Molecular re-analysis at time of progression under TKI-treatment allows to adapt the treatment to the changing molecular profile of the tumor. Liquid biopsy is an evolving, minimally-invasive diagnostic tool, which can detect treatment relevant alterations omitting a re-biopsy in a subset of patients. With the increasing complexity of molecular results, multidisciplinary discussion in molecular tumor boards is recommended to put the findings in the appropriate clinical context and to discuss optimal treatment options.

## 3. Prevalence of Oncogenic Driver Alterations

The prevalence of oncogenic driver alterations depends on clinical variables (e.g., sex, race, smoking status) and tumor-associated factors such as histology and stage [4,46].

In non-squamous histology, the most frequently detected oncogenic driver alterations are KRAS mutations, followed by EGFR mutations, ALK gene rearrangements, BRAF mutations, MET alterations including MET exon 14 skipping mutations and MET amplification, HER2 alterations, RET, and ROS1 gene arrangements and NTRK 1/2/3 gene fusions. While KRAS mutations are the most prevalent oncogenic alterations in metastatic AC (up to 30%), the incidence of EGFR mutations is highly dependent on the tested population (12–47%) [47,48]. Both ALK rearrangements and BRAF mutations account for approximately 5% of all oncogenic alterations in AC. The other oncogenic alterations occur in less than 3% of patients with AC [49,50].

In patients with SCC, the frequency of sensitizing EGFR mutations, ALK, ROS1, and RET rearrangements, MET alterations and BRAF V600 mutations is low and molecular testing not routinely recommended [51,52,53]. The most common genomic alterations found in SCC are FGFR1 amplification (20%) and PI3K aberrations (30–50%) [54].

Molecular oncogenic driver aberrations, their frequency and targeted therapies (in the order discussed below) are summarized in Table 1.

## 4. Overview of Major Oncogenic Driver Alterations and Targeted Agents

A selection of pivotal clinical trials of targeted agents in patients with oncogenic driver alterations is presented in Table 2.

**Figure 1:**
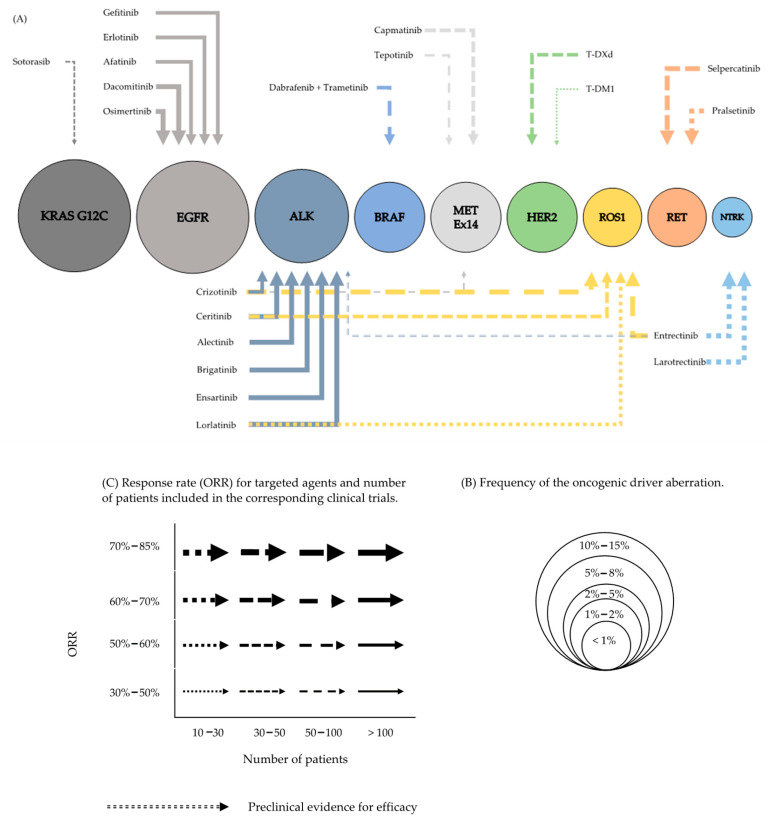
Molecular oncogenic driver aberrations and targeted therapies. Graphic overview (**A**). The size of the circles indicates the frequencies of the oncogenic driver aberration (**B**). The arrow thickness reflects the magnitude of response (ORR) for every targeted agent and the style of the line (continuous, dashed, dotted) the number of patients included in the corresponding clinical trials (**C**).

A graphic overview on molecular oncogenic driver aberrations and targeted therapies is provided in Figure 1.

## 5. KRAS Mutations

KRAS (kirsten rat sarcoma viral oncogene homolog) oncoprotein is a GTPase and an essential mediator of intracellular signaling pathways, including the RAF-MEK-ERK (MAPK) pathway, involved in cell growth and survival [55,56]. KRAS is one of the most commonly mutated genes in human cancers [57]. In NSCLC, KRAS mutations occur in approximately 20–25% of AC and 4% of SCC [4,58]. Contrary to most other oncogenic driver mutations, KRAS mutations are more often found in smokers and less common in East Asian patients [59,60]. In NSCLC, KRAS mutations most often occur in codons 12 and 13 and with a lower frequency in codon 61 [4]. The predominant mutations are G12C, G12V and G12D [61]. The KRAS G12C mutation is present in approximately 13% of patients with NSCLC [57]. KRAS mutations do generally not overlap with other oncogenic mutations. There is increasing evidence that KRAS-mutated AC are heterogenous in their biology and clinical behavior [47]. Based on co-occurring genomic alterations a classification into three subgroups has been proposed: a subgroup with co-occurring TP53-alterations, a second group with co-mutations or genomic loss in STK11 (LKB1) and a subgroup characterized by bi-allelic loss of CDKN2A/CDKN2B locus [62]. This intra-driver heterogeneity possibly explains the different responses to targeted therapy and immunotherapy of KRAS-mutated NSCLC [63].

Although various attempts inhibiting KRAS have been made, there is no established therapy for this large patient subpopulation. So far, the most promising approach was the combination of MEK-inhibitors with chemotherapy [64,65,66]. However, a randomized phase III study combining selumetinib with docetaxel did not confirm the positive phase 2 data [67]. Within the Swiss Group for Clinical Cancer Research (SAKK) network, we have investigated the combination of the MEK-inhibitor binimetinib with cisplatin/pemetrexed as first-line therapy in a phase I/II study. The combination of cisplatin/pemetrexed in combination with binimetinib at 45mg bid was safe with no dose-limiting toxicities (DLT) observed. However, no early signal of increased efficacy of the addition of binimetinib to chemotherapy was observed in purely KRAS-mutant NSCLC [68].

### KRAS G12C Inhibitors

Very recently, encouraging signs of efficacy have been observed for two newly developed specific KRAS G12C inhibitors. In the proximity of the mutated cysteine of the KRAS G12C mutation (glycine-to-cysteine mutation at position 12) there is a pocket (P2) that is present in the inactive GDP-bound conformation of KRAS [69]. Binding of small molecules to the P2 pocket traps KRAS G12C in the GDP-bound inactive form [70,71]. Based on this mechanism of action, specific inhibitors for the KRAS G12C mutation have been or are developed. 

The first KRAS G12C inhibitor that reached clinical testing in humans is sotorasib (AMG 510) [72]. Early clinical trial results from a subset of 34 patients with NSCLC of the phase I CodeBreaK100 trial were presented at the International Association for the Study of Lung Cancer (IASLC) 2019 World Conference on Lung Cancer (WCLC) [73]. Among the 13 patients who received the established target dose of 960 mg and were evaluable for efficacy analysis, seven (54%) achieved a partial response (PR) and six (46%) a stable disease (SD). The final results of the CodeBreaK100 trial investigating sotorasib in different advanced solid tumors have recently been published [74]. In the 59 patients with NSCLC, all pre-treated, the ORR was 32% and therefore lower than previously reported. Median duration of response (mDOR) for NSCLC patients was 10.9 months. Sotorasib was generally well-tolerated and no DLT were observed. The reason for the lower-than-expected ORR observed for sotorasib in NSCLC patients may be explained with co-occurring mutations as mentioned above.

The second KRAS G12C inhibitor that is currently studied in a phase I/II trial (NCT03785249) is adagrasib (MRTX849). The phase I/II KRYSTAL-1 trial included patients with advanced, pretreated solid tumors harboring a KRAS G12C mutation. Overall, 79 patients with KRAS G12C mutant NSCLC have been treated with adagrasib 600 mg bid. Response data are available for 51 patients so far. ORR was 45% and disease control rate (DCR) was 96% [75]. After a median follow-up of 3.6 months, 83% (19/23) of responders have not progressed and remain on study.

Numerous clinical trials with new KRAS inhibitors are currently ongoing. In these trials KRAS G12C inhibitors are compared to conventional chemotherapy or investigated in combination with immune checkpoint inhibitors or other targeted agents.

## 6. EGFR Mutations

Epidermal growth factor receptor (EGFR) belongs to a family of four receptors (EGFR, ErbB2, ErbB3 and ErbB4) and is the best-known and established oncogenic target in advanced and metastatic NSCLC [6,7]. The most common EGFR gene mutations are base-pair deletion at exon 19 (del19) and point mutation at exon 21 (most commonly L858R) [7,76,77]. Both mutations lead to activation of the tyrosine kinase domain and are sensitive to EGFR-TKIs. They are referred to as sensitizing EGFR mutations. EGFR mutations are more frequent in non- or light smokers, patients with adenocarcinoma histology and of East Asian ethnicity [77,78]. 

### 6.1. First-Generation EGFR Inhibitors

#### 6.1.1. Gefitinib

Gefitinib is a first-generation inhibitor of EGFR [79]. After pivotal trials proved strong activity of gefitinib in patients with EGFR mutation-positive (del19, L858R) lung adenocarcinoma [6,7], subsequent phase II trials demonstrated the efficacy of gefitinib in the upfront treatment setting with an ORR of 55% to 75% and a median PFS of 9.2 to 9.7 months [80,81]. Gefitinib was then compared to chemotherapy in three large phase III trials [8,82,83]. 

The landmark Iressa Pan-Asia Study (IPASS) enrolled treatment-naïve Asian patients with stage IIIB or IV lung adenocarcinoma who had never smoked or were light smokers [8]. Presence of an EGFR mutation was not a prerequisite for study inclusion. Patients were randomly allocated to receive gefitinib or carboplatin plus paclitaxel. The study included 1217 patients. In a subgroup analysis of 261 patients with an EGFR mutation (53.6% with del19, 42.5% with L858R), the median PFS was significantly longer among patients receiving gefitinib than among those with carboplatin plus paclitaxel (HR 0.48). Furthermore, the response rate was increased with gefitinib (71% vs. 47%). On the contrary, gefitinib was ineffective in patients with wild-type EGFR. Patients with gefitinib experienced improved quality of life with fewer side effects compared to carboplatin plus paclitaxel.

The WJTOG 3405 study enrolled only patients with EGFR mutations and randomized participants to gefitinib or cisplatin plus docetaxel [82]. Gefitinib was superior to standard chemotherapy in terms of PFS (median, 9.2 vs. 6.3 months) and ORR (62% vs. 32%). An updated analysis reported no statistically significant difference in median OS [84]. The lack of difference in OS outcomes is assumed to be due to a high rate of treatment crossover upon disease progression. Overall, 78 (90.7%) out of 86 patients treated with chemotherapy received gefitinib after progression.

Finally, the NEJ002 trial also exclusively included patients with EGFR mutations [83]. Chemotherapy-naïve patients with advanced NSCLC were allocated to receive gefitinib or carboplatin plus paclitaxel. Patients with gefitinib had a significantly prolonged PFS (median, 10.8 vs. 5.4 months) and an increased response rate (74% vs. 31%). 

#### 6.1.2. Erlotinib

Erlotinib is another first-generation inhibitor of EGFR. Erlotinib has been approved for the treatment of metastatic NSCLC even before the discovery and characterization of the EGFR mutations in 2004. The approval based on the positive results of the phase III BR.21 study [85]. In a then mutation-agnostic setting, erlotinib was investigated against placebo in unselected and pretreated patients with metastatic NSCLC and showed improved PFS and OS.

The identification of the EGFR mutation as a predictive biomarker for EGFR-TKIs led to the initiation of three large phase III studies comparing erlotinib to chemotherapy. All trials demonstrated superiority of erlotinib to chemotherapy in patients with EGFR mutation-positive NSCLC [86,87,88].

The Chinese OPTIMAL study was one of the first trials that compared upfront erlotinib with carboplatin plus gemcitabine in patients with advanced EGFR-mutated NSCLC [86]. Both PFS (median, 13.1 vs. 4.6 months) and ORR (83% vs. 36%) favored erlotinib. The phase III ENSURE trial confirmed these results in patients with EGFR mutation-positive NSCLC from China, Malaysia, and the Philippines [87]. In a European EGFR-mutated population, the efficacy of erlotinib was demonstrated in the EURTAC (European Tarceva versus Chemotherapy) study [88]. First-line erlotinib increased ORR (64% vs. 18%) and prolonged PFS (median, 9.7 vs. 5.2 months) compared to chemotherapy. An OS-benefit was not shown in this trial, likely due to a high crossover rate following disease progression (76% of patients progressing on chemotherapy). 

### 6.2. Second-Generation EGFR Inhibitors

#### 6.2.1. Afatinib

Afatinib is a second-generation TKI that irreversibly inhibits EGFR, ErbB2 and ErbB4 [89]. Afatinib was investigated in the LUX-Lung clinical trial program including eight studies in patients with EGFR-mutated NSCLC in the first-, second- and third-line treatment setting [90,91,92,93,94,95,96,97]. After phase I trials confirmed activity of afatinib in EGFR mutation-positive NSCLC [98,99], the phase II LUX-Lung 2 trial assessed the efficacy of afatinib in patients with lung adenocarcinoma with activating EGFR mutations (exon 18–21) [91]. The response rate to afatinib was 61%. For patients with the most common activating EGFR mutations (del19 and L858R) the ORR was 66%. Afatinib was then compared with platinum-based chemotherapy in a randomized phase III trial (LUX-Lung 3) in patients with treatment-naïve EGFR-mutated AC of the lung [92]. The primary endpoint PFS was prolonged for patients receiving afatinib (median, 11.1 vs. 6.9 months) and a higher response rate was observed with afatinib (56% vs. 23%). Median OS did not differ between the two treatment arms [100]. However, approximately two thirds of patients with progression had crossed-over to the other treatment arm. Finally, Lux-Lung 7 was a large randomized phase IIb trial investigating afatinib to gefitinib in 319 treatment-naive patients with stage IIIB or IV NSCLC harboring a del19 or L858R EGFR mutation [96]. PFS was statistically improved with afatinib (median, 11 vs. 10.9 months), as well as time-to-treatment failure (median, 13.7 vs. 11.5 months) and ORR was higher with afatinib. Grade 3 or higher adverse events in patients receiving afatinib included diarrhea (13%), rash/acne (9%) and fatigue (6%). An updated analysis reported a median overall survival of 27.9 months in patients who had received afatinib compared with 24.5 months in those with gefitinib [101]. 

#### 6.2.2. Dacomitinib

Dacomitinib is a second-generation TKI that irreversibly inhibits ErbB/HER receptors including EGFR, HER 1, HER2 and HER4 [102]. The randomized phase III ARCHER 1050 trial compared dacomitinib versus gefitinib as first-line therapy for patients with EGFR-mutated NSCLC [103]. Patients with brain metastases were excluded from the trial. Median PFS was increased in patients receiving dacomitinib (14.7 vs. 9.2 months). An update of the study reported an OS-benefit for dacomitinib (median, 34.1 vs. 27 months) [104,105]. Dose reductions occurred in 68% of patients receiving dacomitinib. 

### 6.3. Third-Generation EGFR Inhibitors

#### 6.3.1. Osimertinib

About 50–60% of patients treated with first-generation TKIs develop a T790M resistance mutation in exon 20 [106,107]. Thus, efforts have been made to develop therapeutic options to overcome this acquired resistance mechanism. The third-generation EGRF-TKI osimertinib is highly active in NSCLC patients who developed T790M mutation after gefitinib or erlotinib treatment [39]. In the AURA3 trial, osimertinib resulted in an ORR of 71% and a median PFS of 10.1 months. Importantly, osimertinib proved strong intracranial activity. The subsequent FLAURA study included previously untreated patients with locally advanced or metastatic NSCLC harboring a sensitizing EGFR mutation [108]. Patients were randomized 1:1 to receive either osimertinib, or the first-generation TKIs erlotinib or gefitinib. Patients with disease progression under standard TKI were allowed to crossover to osimertinib. PFS, the primary endpoint, was significantly longer in this trial (median, 18.9 vs. 10.2 months). Both, ORR (80% vs. 76%) and mDOR (17.2 vs. 8.5 months) were improved with osimertinib. The final OS analysis was presented at the 2019 European Society of Medical Oncology (ESMO) congress and published thereafter [109]. Median OS was 38.6 months with osimertinib compared to 31.8 months with first-generation EGFR-TKIs. Patients remained longer on osimertinib (70% vs. 47% at 12 months) and time to first subsequent treatment was significantly prolonged with osimertinib (25.4 vs. 13.7 months). Importantly, 30% of patients in both treatment arms received no subsequent anti-cancer therapy. Finally, 30% of patients in the comparator arm crossed over to osimertinib. The results from the FLAURA study established osimertinib as the standard first-line therapy for EGFR-mutated NSCLC.

#### 6.3.2. Other Third-Generation EGFR Inhibitors

Different other third-generation EGFR inhibitors have been developed to target both standard EGFR-sensitizing and T790M resistance mutations, including rociletinib [110], abivertinib [111], lazertinib [112], nazartinib [113] and almonertinib [114]. Efficacy for these EGFR-TKIs was assessed in early phase trials, mostly in heavily pre-treated patients, and the response rates vary considerably. 

### 6.4. Combination Therapies

#### 6.4.1. Combination Therapies with First-Generation TKIs

An interesting therapeutic strategy is the concept of combining EGFR-TKIs with other agents with the aim to achieve better and more durable responses in EGFR-mutated patients. 

The randomized phase II CALGB 30406 trial has been one of the first studies to assess the addition of chemotherapy to erlotinib in EGFR-mutated patients [115]. While efficacy parameters (ORR, PFS, OS) were comparable in the two arms, patient receiving erlotinib plus chemotherapy (carboplatin and paclitaxel) experienced more adverse events compared to erlotinib monotherapy. In another trial, the combination of gefitinib plus chemotherapy (carboplatin and pemetrexed) yielded a higher response rate than gefitinib monotherapy (75% vs. 63%) [116]. Patients experienced a significantly prolonged PFS and OS with the combination, but also more toxicity. Clinically relevant grade 3 or greater toxicities occurred in 51% of patient in the combination therapy arm compared to 25% in the gefitinib-monotherapy arm. In the RELAY study, erlotinib was investigated with or without the anti-VEGF receptor antibody ramucirumab [117]. Again, the combination therapy led to a significantly prolonged median PFS compared to erlotinib plus placebo (19.4 vs. 12.4 months, HR 0.59), at the price of higher toxicity (72% vs. 54% grade 3–4 adverse events). In summary, combination therapies with first-generation TKIs enhance the efficacy at the price of higher toxicity.

#### 6.4.2. Combination Therapies with Third-Generation TKIs

Ongoing randomized clinical trials are investigating the addition of chemotherapy (NCT04035486), bevacizumab (NCT02971501) and ramucirumab (NCT03909334) to the third-generation TKI osimertinib.

#### 6.4.3. Amivantamab and Lazertinib

Interesting results from the CHRYSALIS study (NCT02609776) investigating a combination treatment with amivantamab, a bispecific antibody against EGFR and MET, and the third-generation EGFR-TKI lazertinib, have been presented at the 2020 virtual ESMO congress [118]. This multi-cohort trial included a cohort with 45 patients who had relapsed on osimertinib but were chemotherapy-naïve. With a median follow-up of 4 months the ORR in this group was 36%. In 20 treatment-naïve patients the combination of amivantamab and lazertinib achieved an ORR of 100%. An ongoing phase III trial (MARIPOSA, NCT04487080) is assessing the combination of amivantamab and lazertinib versus osimertinib in previously untreated advanced EGFR-mutated NSCLC. 

### 6.5. EGFR Exon 20 Insertions Mutations

EGFR exon 20 insertions mutations occur in 4–10% of all patients with EGFR mutations. Clinical activity of irreversible EGFR-TKIs in patients with NSCLC harboring EGFR and HER2 exon 20 mutations has been assessed, yielding however disappointing results. The results may be explained due to pharmacokinetics of second-generation EGFR-TKIs as dose-limiting toxicity impeded to attain the inhibitory plasma concentrations of exon 20 insertions. [119]. Patients harboring EGFR exon 20 mutations are generally refractory to standard EGFR-TKIs [120], although osimertinib at a higher daily dose of 160 mg proved moderate clinical activity (ORR 24%) in the phase II ECOG-ACRIN 5162 trial [121]. 

Amivantamab has proven active in the phase I CHRYSTALIS trial, which included pretreated and treatment-naïve patients with advanced NSCLC harboring EGFR exon 20 insertions [122]. Reduction of target lesions was seen in most patients, the ORR was 36% and DCR 67%. 

Recently, data from early phase trial with molecules designed to selectively target EGFR and EGFR2 (HER2) exon 20 insertion mutations have been presented. Mobocertinib (TAK-788), a highly specific TKI, showed anti-tumor activity (ORR 43%) in patients with EGFR exon 20 insertion mutations at the recommended phase II dose (160 mg once daily) [123]. Mobocertinib yielded a median PFS of 7.3 months. Results from the phase II ZENITH20-1 study of poziotinib in previously treated patients with EGFR exon 20 insertions showed an ORR of 14.8% in the intent-to-treat population and 19.3% in the evaluable population [124]. Median PFS was 4.2 months in the intent-to-treat population. No clinical activity (ORR 0%) was detectable for tarloxotinib, another selective EGFR exon 20 inhibitor, which has been evaluated in the RAIN-701 trial [125]. 

### 7. ALK Gene Rearrangements

Anaplastic lymphoma kinase (ALK) is a transmembrane tyrosine kinase receptor that is expressed in neural tissue, the small intestine, and the testes and plays a crucial role in the development of the central nervous system [126]. The ALK receptor is activated after ligand binding to the extracellular receptor domain and dimerization [127]. The echinoderm microtubule-associated protein-like 4 (EML4)–ALK rearrangement arises from an inversion on the short arm of chromosome 2 (Inv(2)) (p21p23)) [127]. Several variants of EML4-ALK have been described that encode the same cytoplasmic tyrosine kinase domain of ALK with different truncations of EML4 [128,129]. Aberrant ALK activation leads to an activation of multiple downstream signaling pathways, primarily the PI3K/mTOR and RAS/RAF/MAPK cascade [130,131,132]. EML4-ALK rearrangement in patients with NSCLC is a relatively rare event, present in approximately 2–8% of NSCLCs [127,133,134], and most commonly seen in younger patients with AC histology and never- or light-smoking history. ALK rearrangements and other oncogenic drivers, such as mutant EGFR and oncogenic RAS, are generally mutually exclusive, consistent with the notion that ALK rearrangement defines a unique molecular subset of NSCLC [135].

#### 7.1. Crizotinib

Crizotinib was the first targeted drug that showed clinical activity in patients with ALK-positive NSCLC with response rates similar to what has been seen in EGFR mutant NSCLC with EGFR-TKIs [136]. The PROFILE 1007 trial was the first randomized phase III trial for ALK-positive patients [137]. In this study, 347 patients with ALK-positive NSCLC, previously treated with chemotherapy, were randomized to crizotinib or chemotherapy (pemetrexed or docetaxel). ORR for crizotinib was 65% compared to 20% in the comparator arm. The primary endpoint of PFS was prolonged from 3 months with chemotherapy to 7.7 months for patients treated with crizotinib. Furthermore, the PROFILE 1014 trial included 343 chemotherapy-naïve ALK-positive patients randomized to crizotinib or platinum-based chemotherapy [138]. PFS was significantly longer with crizotinib (median, 10.9 vs. 7.0 months). Crossover was allowed in this trial which likely explains the lack of OS benefit observed for crizotinib (not reached vs. 47.5 months, HR 0.76, *p* = 0.0978) [139]. 

Although crizotinib yields high response rates, durable responses are rare and most patients eventually relapse. This led to the development of more specific ALK inhibitors that were able to overcome crizotinib-resistance.

#### 7.2. Ceritinib

Ceritinib is an inhibitor of ALK and ROS1 and demonstrated activity in patients with ALK-rearranged NSCLC who had progressed on crizotinib. In the phase II ASCEND-2 trial, patients who have been previously treated with at least one platinum-based chemotherapy and progressed on crizotinib achieved an ORR of 38.6% [140]. The duration of response was 9.7 months. Common adverse events included nausea (81.4%), diarrhea (80.0%), and vomiting (62.9%). In the ASCEND-4 trial, ceritinib was compared to platinum-based chemotherapy as first-line therapy [141]. Ceritinib improved PFS by 8 months (median, 16.6 vs. 8.1 months). The ASCEND-8 trial assessed whether a lower dose of ceritinib (450 mg or 600 mg, taken with a low-fat meal) improved gastrointestinal tolerability compared to the standard dosing [142,143]. The ORR in the three arms (450 mg fed/600 mg fed/750 mg fasted) were comparable (72–78%). Although gastrointestinal toxicity was the lowest in the 450mg-arm, the frequency remained high (75.9%). Finally, ceritinib has not been compared to other ALK-TKIs. 

#### 7.3. Alectinib

Alectinib is a highly selective ALK inhibitor [144,145] and has been compared in three randomized phase III studies to crizotinib. In the phase III J-ALEX trial, 207 Japanese patients with treatment-naïve ALK-positive NSCLC have been randomized to alectinib in a lower than standard dose of 300 mg bid or crizotinib. Median PFS for alectinib-treated patients was 34.8 months versus 10.9 months with crizotinib [146]. In this study crossover was allowed. HR for OS was 0.80. In the international randomized phase III ALEX trial, alectinib (600 mg bid) was compared to crizotinib in 303 patients with treatment-naïve ALK-positive NSCLC [147]. PFS, the primary endpoint of the trial, was found to be significantly higher with alectinib compared to crizotinib (HR 0.47). Updated results confirmed the significant improvement in PFS [148]. Median PFS with alectinib was 34.8 months compared to 10.9 months with crizotinib. The median OS with alectinib treatment was still not reached in an updated analysis in 2020 and the 5-year OS rate was 62.5% [149]. CNS progression with alectinib was lower compared to crizotinib (12% vs. 45%). Objective responses were achieved in 83% of patients in the alectinib group, versus 76% with crizotinib. Alectinib had a more favorable safety profile than crizotinib (41% vs. 50% grade 3 to 5 adverse, respectively). These results were confirmed in the Asian ALESIA study with a significant overall survival benefit for alectinib [150]. 

#### 7.4. Brigatinib

Brigatinib is an ALK inhibitor that targets ALK mutations, ROS1 rearrangements, and has preclinical activity against EGFR [151,152,153]. In the phase II ALTA study, 222 pretreated ALK-positive patients received brigatinib at two-dose levels (90 mg once daily or 180 mg once daily) [154]. ORR was 45% in arm A (90 mg once daily) and 54% in arm B (180 mg once daily). A high intracranial response rate was observed (42% in arm A and 67% in arm B). Median PFS was 9.2 and 12.9 months in arms A and B, respectively. The phase III ALTA-1L trial, assessed brigatinib versus crizotinib as upfront therapy for patients with ALK-positive NSCLC who had received not more than 1 prior systemic treatment line [155]. Objective responses were achieved in 74% of patients with brigatinib and in 63% with crizotinib [156]. Intracranial responses were higher with brigatinib (66%) compared to crizotinib (16%). Median PFS for patients receiving brigatinib was 24 months compared to 11 months in the crizotinib-arm. 

#### 7.5. Lorlatinib

Lorlatinib is a potent third-generation inhibitor of ALK and ROS1 tyrosine kinases [157]. In phase II trial, lorlatinib has shown activity in ALK-TKI pretreated patients with significant response for brain metastases [158]. Patients had received at least one previous ALK inhibitor. Lorlatinib led to an ORR of 47%. In patients with measurable baseline CNS metastases, an objective intracranial response of 63% was achieved with grade 3 to 4 adverse events including hypercholesterolemia (16%), hypertriglyceridemia (16%) and central nervous system affection (cognitive effects, 1%). First-line lorlatinib has been compared to crizotinib in the randomized phase III CROWN study (NCT03052608) which included 296 previously untreated patients with ALK-positive stage IIIB/IV NSCLC. The first results from a planned interim analysis have been presented at the virtual ESMO congress 2020 [159]. Lorlatinib significantly improved PFS compared with crizotinib. At data cut-off the median follow-up was 18.3 months for lorlatinib and 14.8 months for crizotinib and median PFS times for lorlatinib and crizotinib were not reached and 9.3 months (HR 0.28), respectively. Lorlatinib demonstrated high intracranial activity (intracranial ORR of 82% in patients with measurable brain metastases with 71% complete responses). Responses seemed to be durable. The incidence of grade 3/4 adverse events was higher with lorlatinib (72.5%) than crizotinib (55.6%), mainly due to laboratory abnormalities, especially hypercholesterinemia and hypertriglyceridemia. Serious adverse events occurred at a rate of 34% with lorlatinib, but less than 10% of patients discontinued lorlatinib due to adverse events. 

#### 7.6. Ensartinib

Ensartinib, a new-generation ALK inhibitor, was investigated in 160 patients with ALK-positive metastatic NSCLC who had progressed on crizotinib therapy [160]. Response assessment was performed in 147 patients and ORR was 52%. Among patients with measurable brain metastases, the intracranial response rate was 70%. 

Interim results from the phase III eXalt3 study have been presented at the 2020 IASLC’s World Conference on Lung Cancer Virtual Symposium [161]. The trial included 290 patients with ALK-positive stage IIIB or IV disease who had not previously received an ALK inhibitor and no more than one prior line of chemotherapy. Participants were randomized to receive ensartinib or crizotinib. With a median follow-up of 23.8 (ensartinib) and 20.2 months (crizotinib) PFS was significantly prolonged for patients assigned to receive ensartinib compared to those receiving crizotinib (median, 25.8 vs. 12.7 months). Both the ORR and the intracranial ORR in patients with baseline brain metastases were higher with ensartinib compared to crizotinib (75% vs. 67% and 64% vs. 21%, respectively). Overall survival data were immature. Adverse events were more frequent with ensartinib. 

#### 7.7. Entrectinib

Entrectinib is an inhibitor of ALK, ROS1 and pan-TRK with activity in ALK-positive NSCLC in early phase studies [162]. Entrectinib is currently investigated in a randomized study versus crizotinib in the first-line treatment setting of ALK-positive NSCLC (NCT02767804). 

## 8. BRAF Mutations

v-Raf murine sarcoma viral oncogene homolog B (BRAF) is a serine/threonine kinase that is part of the MAP/ERK signaling pathway. Activating point mutations in BRAF result in unregulated signaling via the MAP/ERK pathway. BRAF mutations have initially been described in malignant melanoma with 40–60% of tumors harboring an activating BRAF V600E mutation [163]. Subsequently, BRAF mutations have also been detected in colorectal cancer, papillary thyroid cancer and other solid tumors [163,164,165]. Somatic BRAF mutations occur in 1–5% of lung AC with BRAF V600E representing approximately half of all BRAF mutations [166,167]. Other mutations occur within exons 11 and 15 [167]. The novel classification of BRAF mutations identifies three distinct functional classes: class I, including RAS-independent kinase-activating V600 functioning as monomers; class II, RAS-independent kinase-activating nonV600 dimers; and class III, RAS-dependent kinase-inactivating nonV600 heterodimers [168,169]. BRAF V600E mutations are associated with light/never smoking status, micropapillary histology and occur more frequently in female patients. In contrary, non-V600E mutations are more frequent in former or current smokers and are associated with poorer outcome [167,170]. Generally, patients with BRAF mutation positive NSCLC have a lower response rate to first-line platinum-based chemotherapy and a poor survival [167,171,172].

The presence of the BRAF V600E mutation is associated with response to BRAF as well as combined BRAF and MEK inhibitors. 

### 8.1. BRAF Inhibitors

BRAF inhibitors have successfully been investigated in BRAF-mutant malignant melanoma [173]. In 2015, the results of the European EURAF registry study were published [174]. In 35 patients, the ORR with BRAF therapy was 53% and DCR was 85%. Subsequently, single agent dabrafenib was tested in patients with BRAF V600E mutated NSCLC [175]. The ORR was 33% with a DCR of 57% in the pretreated group of patients. Median PFS was 5.5 months. Another BRAF inhibitor, vemurafenib was tested as a monotherapy in a basket study of BRAF V600E non-melanoma cancers [176]. Twenty patients with BRAF V600E mutated NSCLC were included in this study. The ORR was 42% and median PFS was 7.3 months.

### 8.2. Dabrafenib and Trametinib

The efficacy of combined treatment with the BRAF inhibitor dabrafenib and the MEK 1/2 inhibitor trametinib in patients with metastatic BRAF V600E-mutated NSCLC has been demonstrated in an open-label phase II trial (NCT01336634) including previously pre-treated (cohort B) and untreated patients (cohort C). Thirty-six of 57 pre-treated patients (63%) receiving the combination of dabrafenib/trametinib achieved an investigator-assessed objective response [177]. In an updated analysis the (investigator-assessed) ORR was 68% [178]. In treatment-naïve patients the investigator- and independent review committee-assessed ORR was 64% [179]. Median investigator-assessed PFS was 10.2 months for pre-treated and 10.8 months for untreated patients [178]. Median PFS assessed by blinded independent central review (BICR) was 14.6 months in the previously untreated patients. Patients in the treatment-naïve population had a median OS of 17.3, while it reached 18.2 months in the pre-treated cohort of patients. Serious adverse events (grade 3–4) occurred in 69% and 56% of patients, respectively, including pyrexia (11–16%), hypertension (11%) and elevated liver enzymes (11%). Results from these studies have been subsequently confirmed in a real-world setting [180]. Clinical trials currently underway are investigating the efficacy of other BRAF and MEK inhibitors, for example encorafenib and binimetinib (NCT03915951). 

### 8.3. Non-V600 Mutations

No data exists to support the use of BRAF/MEK inhibitors for non-V600 mutations and chemotherapy or immunotherapy remain the preferred treatment options. Outcome and response rate to Vemurafenib-monotherapy in patients with non-V600 BRAF mutated NSCLC has been disappointing [181]. 

## 9. HER2 Alterations

Human epidermal growth factor receptor 2 (HER2), also known as ErbB2, is a member of the ErbB receptor tyrosine kinase family. There is no known HER2 ligand and activation is induced by homo- or heterodimerization [182,183]. Activating mutations in HER2 are detected more commonly in adenocarcinoma histology and never smokers [184]. They can be found in up to 6% of tumors lacking EGFR, KRAS, and ALK alterations, since these are mutually exclusive with HER2 mutation [185]. The most common mutation is a 12-base pair insertion in exon 20. Amplification of HER2, detected by fluorescence in situ hybridization (FISH), occurs in 2–4% of patients and at a higher rate as resistance mechanisms to EGFR-TKIs [186]. Protein overexpression by immunohistochemistry (IHC 2+ or 3+) is detected in 13–20% of patients, although strong expression is only found in 2–4% [187,188]. In contrast to breast cancer, there is a lack of correlation between HER2 amplification, HER2 mutations and protein overexpression [189], which renders the assessment of efficacy of anti-HER2 agents in various trials difficult.

### 9.1. Trastuzumab-Based Treatment

In the retrospective European EUHER2 cohort study, the ORR of the pooled TKIs (afatinib, lapatinib and neratinib) was 7.4%, while the response rate with trastuzumab-chemotherapy combinations or ado-trastuzumab emtansine was 50.9% [190]. Other phase II trials confirmed the efficacy of trastuzumab-chemotherapy combinations [191,192]. However, the additional benefit attributed to trastuzumab in the combinations with chemotherapy appeared to be limited [193]. 

### 9.2. Antibody-Drug Conjugates

Ado-trastuzumab emtansine (T-DM1) is an antibody-drug conjugate (ADC) composed of trastuzumab linked to DM1, a cytotoxic microtubule-inhibitory agent. T-DM1 proved effective in a single-center phase II basket trial with HER2-mutated adenocarcinoma, demonstrating an investigator-assessed ORR of 44% and median PFS of 5 months [194]. Responses were seen across all HER2 mutation subtypes. In a recent analysis of this study including an additional expansion cohort, the results of a total of 49 patients with HER2-amplified and/or -mutated tumors were reported [195]. The ORR was 50% in both cohorts. T-DM1 was also tested in patients with HER2-positive NSCLC selected by immunohistochemical protein-overexpression (HER2 IHC 2+ or IHC 3+) [196]. Only limited activity was observed (ORR of 20% in IHC 3+, 0% in IHC 2+), illustrating the limitation of HER2 status assessment by immunohistochemistry. Another phase II trial assessed T-DM1 in NSCLC patients with HER2 IHC 3+ or IHC 2+ and FISH positivity or exon 20 mutation [197]. This trial was terminated early due to limited efficacy. 

At the 2020 American Society of Clinical Oncology (ASCO) congress, results from the DESTINY-Lung 01 trial, a multicenter, phase II study, investigating the novel ADC trastuzumab deruxtecan (T-DXd) in patients with HER2-mutated NSCLC have been presented [198]. T-DXd is composed of the anti-HER2 antibody trastuzumab, a cleavable linker, and a topoisomerase I inhibitor. In the cohort of NSCLC patients with HER2-mutations, T-DXd resulted in an ORR by BICR of 62% and a median PFS of 14 months. The treatment was well-tolerated with fatigue (11.9%) and nausea (9.5%) being the most common adverse events associated with dose reduction. As an adverse event of special interest there were cases of interstitial lung disease (11.9% G2, no G3/4 cases). Similar results have been presented from a phase I study including 18 patients with HER2-overexpressing and/or HER2-mutant NSCLC [199]. ORR was 55.6% and the median PFS was 11.3 months. Higher activity was seen among 11 patients with HER2 mutation positive disease with an ORR of 72.7%. Four patients experienced interstitial lung disease or pneumonitis and one patient died due to respiratory failure related to T-DXd. T-DXd is currently being investigated in combination with pembrolizumab (NCT04042701) in patients with NSCLC harboring either a HER2 overexpression or a HER2 mutation. 

### 9.3. Pan-HER2 TKIs

Different pan-HER2 TKIs have been evaluated in HER2-altered NSCLC. Afatinib, a TKI with activity against ErbB family members, is approved for EGFR-mutated AC, but has not shown meaningful activity in lung cancer patients harboring HER2-mutations [200]. Neratinib, an irreversible pan-HER inhibitor, has failed to demonstrate relevant activity in a basket trial including a cohort with HER2-mutated NSCLC patients [201]. Likewise, dacomitinib, a pan-HER2 inhibitor, has not proven very effective with an ORR of 12% in a phase II trial with HER2-mutated NSCLC patients [202]. In contrary, pyrotinib, an irreversible pan-HER2 TKI, has shown higher effectivity [203]. In a multicenter phase II study conducted in China, pyrotinib resulted in an ORR assessed by independent central review of 30% with a median DOR of 6.9 months. Diarrhea (20% grade 3/4), nausea and vomiting were commonly seen adverse events. 

### 9.4. Selective Inhibitors of HER2 exon 20 Insertions

Interesting results have been recently presented regarding TKIs that selectively target both EGFR and HER2 exon 20 insertion mutations, including mobocertinib, poziotinib, and tarloxotinib. Mobocertinib has been assessed in a phase I/II trial (NCT02716116), including a cohort of patients with HER2 exon 20 mutated NSCLC. The results have not yet been published. Results from the multi-cohort phase II ZENITH20 trial of poziotinib in patients with EGFR or HER2 exon 20 mutations have been presented at the virtual 2020 ESMO congress [204]. The results originated from the cohort of patients with previously treated HER2 exon 20-mutated NSCLC. The ORR was 27.8% in all 90 patients and 35.1% in the 74 evaluable ones. Poziotinib showed a manageable safety profile with diarrhea (26%), rash (30%) and stomatitis (22%) as most common grade 3 adverse events. First results of the RAIN-701 trial assessing the hypoxia activated pan-HER inhibitor tarloxotinib in NSCLC and other solid tumors have been presented at the virtual 2020 ESMO congress [125]. Among the 9 evaluable patients with HER2 exon 20 insertion mutated NSCLC, the ORR was 22%. Tarloxotinib showed a favorable safety profile with grade 3 rash or diarrhea in less than 5% of patients. 

## 10. ROS1 Gene Rearrangements

The c-ros oncogene 1 (ROS1) encodes a tyrosine kinase receptor from the insulin receptor family. A rearrangement of ROS1 has initially been described in glioblastoma [205,206,207]. In 2007, ROS1 rearrangement was found in NSCLC cell lines and primary tumors [208]. ROS1 fusion partners include SLC34A2, CD74, TPM3, SDC4, EZR, LRIG3, KDELR2, and CCDC6 [129]. ROS1 rearrangement has been described in 0.7–1.7% of NSCLC patients [129,209]. Similar to previously described oncogenic aberrations in lung cancer, ROS1 translocation is predominantly found in younger patients with AC histology who are either never, or former light smokers [210]. 

### 10.1. Crizotinib

The phase I PROFILE 1001 study investigated crizotinib, a tyrosine kinase inhibitor of ALK, ROS1 and MET, versus platinum-based chemotherapy in patients with ALK-positive NSCLC [138]. An expansion cohort of this trial included patients with ROS1 rearranged NSCLC [211]. Crizotinib demonstrated a very effective and durable anti-tumor activity (ORR 72%, DOR 17.6 months) [211]. Updated results reported an even prolonged DOR (24.7 months), mOS was 51.4 months and 4-year OS rate reached 51% [212]. The robust anti-tumor activity has been confirmed in two prospective phase II studies (ORR 70% and 69%, respectively) [213,214] and in the retrospective EUROS1 study (ORR 80%) [215]. 

### 10.2. Ceritinib

Ceritinib, an ALK and ROS1 inhibitor, was investigated in a Korean phase II study, where 32 patients with ROS1-rearranged advanced NSCLC were treated [216]. The ORR reached 62% in the 28 patients with response-evaluable disease. For 20 patients without prior Crizotinib-therapy the response rate was 67%. The median PFS was 9.3 months for all patients and 19.3 months for crizotinib-naïve patients.

### 10.3. Lorlatinib

Activity against ROS1 has also been described for lorlatinib [217]. In 69 ROS1-positive patients, including 40 crizotinib-pretreated patients, 41% had an objective response. Among the TKI-naïve patients, the ORR was 62%. The intracranial response rate was 50%. 

### 10.4. Entrectinib

For entrectinib, a multikinase inhibitor, a pooled analysis from three phase I-II trials (STARTRK-2, STARTRK-1, and ALKA-372-001) showed an impressive ORR of 77% and median PFS of 19.0 months [218]. An intracranial activity of 55% was reported for patients with baseline CNS metastases. Grade 3 to 4 adverse events were seen in 34% of patients.

### 10.5. Repotrectinib and Taletrectinib

Repotrectinib and Taletrectinib are two new ROS1 inhibitors and have been evaluated in early phase studies for ROS1-positive NSCLC.

The ongoing TRIDENT-1 study investigates repotrectinib in patients with advanced ROS1-, TRK-, and ALK-positive solid tumors. First safety and efficacy signals in the cohort of patients with ROS1 positive NSCLC were published in 2019 [219]. ORR was 90% among the 10 TKI-naïve patients and 28% among the 18 TKI-pretreated patients. Treatment was well-tolerated.

Taletrectinib is an orally available and potent selective small molecule inhibitor of ROS1 and NTRK with activity in NSCLC patients with ROS1 fusion [220]. In a phase I study in patients with advanced solid tumors, the ORR among the six patients with crizotinib-refractory NSCLC was 33.3% [221]. A phase II trial investigating the efficacy of taletrectinib in patients with TKI-naïve and crizotinib-pretreated ROS1-postive NSCLC is currently ongoing in China (NCT04395677). 

## 11. RET Gene Rearrangements

Rearranged during transfection (RET) is a receptor tyrosine kinase and a known oncogene in thyroid cancer, where translocations, as well as activating mutations, have been detected [222,223]. The most common RET alterations in NSCLC are gene rearrangements (fusions) between the RET gene and other partners, the most common being kinesin family member 5B gene (KIF5B) [27]. They are found in 1–2% of lung cancers (mostly AC) and are mutually exclusive with other oncogenic drivers [224]. RET fusions are more common in younger patients and never- or light-smokers and associated with solid (with or without signet ring cells), papillary and lepidic patterns [225,226]. RET-positive NSCLC often manifests with rather small primary tumors with little or no lymph node involvement, but with a high prevalence of pleural dissemination and brain metastases [227,228]. Despite these clinically unfavorable factors, RET fusion appears to be associated with an overall more favorable prognosis [229].

Patients with RET-fusion positive NSCLC show a high response to pemetrexed-based chemotherapy regimens, possibly due to a lower expression of thymidylate synthetase in the tumor tissue [230,231].

### 11.1. Non-Selective RET Inhibitors

Different non-selective RET inhibitors have been assessed retrospectively in a large international registry of patients with NSCLC harboring RET-fusions [232]. In this trial, the response rates for cabozantinib, vandetanib, and sunitinib were 37%, 18%, and 22%, respectively. The limited clinical activity was confirmed for cabozantinib [233,234] and vandetanib [235] in prospective clinical trials leading to an ORR of 28% and 18%, respectively. In a small Japanese trial with 19 pre-treated patients the ORR for vandetanib was higher (47%) [236]. Both cabozantinib and vandetanib were associated with a high rate of adverse events requiring dose reductions. Lenvatinib, another TKI, yielded only limited clinical activity (ORR 16%) in a phase II trial [237]. Alectinib showed response in patients with pretreated RET-positive NSCLC in two small case series [238,239]. The phase I/II ALL-RET study failed to demonstrate convincing activity of alectinib [240]. The dose selected on the phase I study was 450 mg bid. Among 25 patients, only one patient had a response. The DCR was 52% and median PFS was 3.4 months. The European ALERT-lung trial led by ETOP is still recruiting (NCT03445000). In this trial, patients with proven RET fusion and at least one prior treatment with platinum-based chemotherapy will be treated with alectinib 600 mg bid.

### 11.2. Agerafenib

The safety and antitumor activity of the multikinase RET inhibitor agerafenib (RXDX-105) has been explored in a phase I/Ib trial [228]. ORR in RET inhibitor-naïve patients (cohort Ib) was 19% (6/31). Interestingly however, the ORR varied significantly by the gene fusion partner. The ORR was 0% for patients with tumor harboring KIF5B as fusion partner with RET, compared to 67% with non-KIF5B partners. 

### 11.3. Selpercatinib and Pralsetinib

High efficacy has been demonstrated with two new selective RET inhibitors: selpercatinib (LOXO-292) and pralsetinib (BLU-667).

Selpercatinib, has been investigated in the phase I/II Libretto-001 study, with a cohort of 531 patients with RET fusion-positive NSCLC [241,242]. Among the primary analysis set of patients with prior platinum-based treatment, the ORR of selpercatinib was 65% as determined by BICR, whereas treatment-naïve patients had an ORR of 85%. Responses were observed regardless of RET fusion partner. The median PFS was 16.5 months for pre-treated patients and not reached for those without previous treatment. Selpercatinib-therapy was well-tolerated. Only 2% of patients discontinued treatment due to adverse events. Selpercatinib showed high intracranial response (81.8%) with a median duration of intracranial response of 9.4 months [243]. Selpercatinib is currently investigated in a randomized phase III study versus standard first-line chemo-immunotherapy (LIBRETTO-431, NCT04194944).

Pralsetinib (BLU-667) is investigated in the multi-cohort phase I/II ARROW trial (NCT03037385) in patients with RET gene fusion-positive tumors including NSCLC patients. The first results were presented at the 2019 ASCO annual meeting [244]. The registrational dataset was recently presented [245]. Efficacy for RET fusion-positive NSCLC was evaluated in 80 chemotherapy pre-treated patients. Pralsetinib demonstrated an ORR of 61%. In 26 treatment-naïve patients the response rate was 73%. Treatment was well-tolerated with low-grade and reversible side effect, including increased aspartate aminotransferase (31%), anemia (22%), increased alanine aminotransferase (21%), constipation (21%) and hypertension (20%). Only 4% of patients discontinued treatment due to a drug-related adverse event. Finally, pralsetinib has demonstrated central nervous system activity. 

## 12. MET Alterations

MET is a proto-oncogene encoding a receptor tyrosine kinase (c-MET), which undergoes homodimerization by binding its ligand, hepatocyte growth factor (HGF). Homodimerization and autophosphorylation of c-MET leads to the activation of various intracellular signaling pathways including RAS-RAF-MAPK, JAK-STAT and PI3K-AKT-mTOR [246]. Gain-of-function alterations of MET can occur by gene amplification or by MET exon 14 skipping mutations, which impairs degradation of c-MET receptors [28]. c-MET can also be transactivated in a ligand-independent manner by ErbB/HER family receptors [247]. In NSCLC, MET exon 14 skipping mutations occur in 2–4% of patients with AC, but have been described in up to 30% of pulmonary sarcomatoid carcinoma [248,249]. MET exon 14 skipping mutations are more frequent in former smokers, female and older patients (median age > 70 years) [28,248]. Patients with MET exon 14 mutations have a worse prognosis compared to wild-type patients [249,250,251]. The prevalence of MET amplification is reported to be 4–5% [249,252]. However, MET-amplification occurs much more frequent as a resistance mechanism, for example in EGFR-mutated AC treated with EGFR-TKIs [253].

First therapeutic approaches for MET-altered NSCLC included combination treatments of MET inhibitor tivantinib with erlotinib [254] and monoclonal MET antibody onartuzumab with erlotinib [255]. Both combinations had only limited activity.

### 12.1. Crizotinib

Crizotinib, a non-selective MET inhibitor, has demonstrated clinical efficacy both for MET amplifications and MET exon 14 mutations. 

In the phase I/II PROFILE 1001 trial, 69 patients with tumors harboring MET exon 14 alterations were included [256]. Most of the included patients (62%) had received more than one previous treatment line. The ORR for crizotinib in 65 evaluable patients was 32% and the median PFS was 7.3 months. While CNS metastases have been described to occur in less than 20% of patients with MET exon 14 altered NSCLC at diagnosis, the incidence approximately doubles during the course of the disease [257]. In patients with crizotinib-treatment, CNS progression was described in 24% of patients [257]. 

Responses to crizotinib have also been observed in patients with MET-amplified tumors [36,214,258]. However, the ORR varied considerably depending on the MET-amplification level. Furthermore, there was inconsistency regarding the definition of MET-amplification across the trials investigating crizotinib. In Camidge et al., tumors were categorized into a low (MET/CEP7 ratio ≥1.8–≤2.2), intermediate (>2.2–<5) and high amplification level (≥5) [258]. For the 6 patients with high-level MET amplification the ORR was 50%.

### 12.2. Capmatinib

Capmatinib, a selective MET inhibitor, has been evaluated in the multi-center, multi-cohort phase II GEOMETRY mono-1 study [259]. GEOMETRY mono-1 enrolled patients with advanced NSCLC with a MET exon 14 mutation and/or a MET amplification. The trial comprised 7 cohorts: 5 cohorts for assessment of efficacy and 2 expansion cohorts. Patients who had received prior treatment were enrolled to cohorts 1–4 and 6, while those who were treatment-naïve were enrolled to cohorts 5a, 5b and 7.

In treatment-naïve patients with NSCLC bearing MET exon 14 mutations (cohort 5b), capmatinib demonstrated an ORR of 68%. Median DOR was 12.6 months and mPFS 12.4 months. Patients with MET exon 14 mutations who had prior treatment (cohort 4) had an ORR of 41%, mDOR of 9.7 months and mPFS of 5.4 months. Results from the expansion cohort of pre-treated patients with MET exon 14 mutations (cohort 6) were in line with the efficacy of capmatinib in cohort 4 (ORR 48%). The intracranial activity in patients with NSCLC harboring MET exon 14 mutations (cohort 4 and 5b) was 54%. Activity of capmatinib was also reported in patients with high-level MET-amplified metastatic NSCLC (gene copy number ≥10), although response rates were lower than in patients with MET exon 14 mutations. In treatment-naïve patients the observed ORR was 40% (cohort 5a), and 29% in pre-treated patients (cohort 1a). Median DOR in these patients was 7.5 and 8.3 months, respectively. 

Most common treatment-related adverse events across all cohorts were peripheral edema (51% all-grade and 9% grade 3–4 AEs), nausea (45% and 2%), and vomiting (28% and 2%). Laboratory alterations included increased blood creatinine in 24% of patients. 

### 12.3. Tepotinib

Tepotinib is another small molecule MET inhibitor. VISION, a multi-center, multi-cohort phase II trial, assessed tepotinib in patients with locally advanced or metastatic NSCLC with MET exon 14 skipping mutation and MET amplifications [260]. Among 152 patients with MET exon 14 skipping mutations treated with tepotinib, 99 were included in the efficacy population defined as patients with a follow-up of at least 9 months. Efficacy and outcome parameters were only reported for this population. The ORR was 46%, mDOR reached 11.1 months and median PFS was 8.5 months. Tepotinib had activity in patients with brain metastases (intracranial ORR 55%). The safety profile of tepotinib was comparable to capmatinib. The results for the patient-cohort with MET-amplified NSCLC have not been presented yet.

### 12.4. Savolitinib

Savolitinib, another selective MET inhibitor, has demonstrated durable clinical activity in patients with NSCLC who harbor a MET exon 14 skipping mutation [261]. Results of a Chinese phase II trial (NCT02897479) have been presented at the 2020 ASCO virtual meeting. Savolitinib led to an ORR of 42.9%, as assessed by an independent review committee, and a median DOR of 9.6 months. The most common grade 3 or higher adverse events included peripheral edema (7.1%) and increase of ALAT (10%) and ASAT (12.9%).

### 12.5. Future Direction

Interesting early results have been shown for Sym015, a synergistic mixture of two recombinant monoclonal antibodies against non-overlapping epitopes of MET [262]. In the phase IIA Sym015-01 trial, 20 patients with NSCLC (MET-amplification or MET exon 14 mutations) received Symp015 and the ORR was 25%. Response rates for treatment-naïve patients were comparable to other MET-TKIs. 

Amivantamab (JNJ-61186372), a bispecific EGFR-MET antibody is tested in the CHRYSALIS phase I study (NCT02609776). This study comprised a dose expansion phase in patients with EGFR- and MET-mutated NSCLC.

Telisotuzumab vedotin (ABBV-399) is an ADC targeting c-MET and is currently tested in phase II study (NCT03539536).

## 13. NTRK Gene Fusions

Neurotrophic tyrosine receptor kinase (NTRK) genes encode tropomyosin receptor kinase (TRK) fusion proteins that act as oncogenic driver in different types of tumors [263]. Numerous fusion partners have been identified [264]. NTRK fusions are frequent in some rare cancer types (e.g., secretory breast carcinoma and congenital fibrosarcoma), but rarely occur in other solid tumors with a prevalence of 0.1–1% in unselected NSCLC [265]. The prevalence is higher (up to 3%) in tumors lacking KRAS, EGFR, ALK and ROS1 alterations [266]. Different multikinase agents display some activity against TRK (e.g., cabozantinib, crizotinib, nintedanib), but the most potent TRK-inhibition has been shown for the first-generation TRK tyrosine kinase inhibitors larotrectinib and entrectinib. Larotrectinib (LOXO-101) and entrectinib (RXDX-101) have both been tested in a tumor-agnostic setting. 

### 13.1. Larotrectinib

Larotrectinib has been assessed in an adult phase I trial, a pediatric phase I/II trial (SCOUT) and an adult/adolescent phase II basket trial (NAVIGATE) [267]. The ORR for larotrectinib reported in the first publication in 55 patients with NTRK gene fusion-positive disease across a wide range of tumors was 75%. Of note, this study included patients with 17 different malignancies and only 4 patients with NSCLC. In a recent update, results from a pooled analysis of 159 patients (including 12 patients with lung cancer) were presented [268]. In the overall population the ORR for larotrectinib was 79% and 73% in adult patients. Tumor response was seen irrespective of tumor type. In lung cancer patients, the ORR based on RECIST criteria was 75% (9/12). In the overall population, mPFS was 28.3 months. Larotrectinib showed good tolerability, with only very few serious adverse events (anemia, liver enzyme elevation, fatigue) and a low rate of patients who discontinued (2%) or reduced the dose (8%) of the drug due to treatment-related adverse events.

### 13.2. Entrectinib

Entrectinib was tested in an adult phase I trial (ALKA-372-001), an adult phase I trial (STARTRK-1), a phase II basket trial (STARTRK-2), and a phase I/II pediatric trial (STARTRK-NG) [162,269,270]. A pooled analysis of these trials reported an ORR of 57% [271]. The mDOR was 10 months and mPFS 11.2 months. Again, response occurred regardless of tumor type. In 10 patients with NSCLC the ORR was 70%. Similar to larotrectinib, entrectinib showed a favorable safety profile. 

### 13.3. Future Direction

Currently, second-generation NTRK inhibitors, such as repotrectinib (NCT04094610, NCT03093116) and selitrectinib (NCT03215511), are investigated in clinical studies.

## 14. Conclusions

Recent developments in the subgroup of NSCLC with oncogenic driver alterations, highlighted in the present review, have brought significant clinical patient benefit and established an individualized treatment approach. The treatment landscape of oncogenic-addicted NSCLC is becoming increasingly complex. The choice of the optimal treatment strategy and management of TKI-therapy, including adverse events, requires expertise and a multidisciplinary approach. Whilst further progress is expected, the prerequisite for this is an ongoing effort to include patients in clinical trials.

## Figures and Tables

**Table 1:** Molecular oncogenic driver aberrations, their frequency and targeted therapies.

Genomic Driver Alterations	Most Common Subtype	Frequency ^#^	Investigated Targeted Agents
KRAS mutations	G12C, G12V, G12D	Adenocarcinoma: 20–25% Squamous cell carcinoma: 4% KRAS G12C: 13%	KRAS G12C inhibitors: Sotorasib, Adagrasib
EGFR mutations	Deletion 19, L858R	12–15%	First-generation EGFR inhibitors: Erlotinib, Gefitinib Second-generation EGFR inhibitors: Afatinib, Dacomitinib Third-generation EGFR inhibitor: Osimertinib
ALK gene rearrangements	EML4-ALK fusion	2–8%	First-generation ALK inhibitor: Crizotinib Second-generation ALK inhibitors: Ceritinib, Alectinib, Brigatinib Third-generation ALK inhibitor: Lorlatinib ALK, ROS1 and pan-TRK inhibitor: Entrectinib
BRAF mutations	V600E	1–5% (50% V600E)	BRAF inhibitor: Dabrafenib MEK inhibitor: Vemurafenib BRAF + MEK inhibition: Dabrafenib + Trametinib
HER2 alterations	HER2 amplification HER2 Exon 20 mutation	2–4% 1–2%	Trastuzumab + chemotherapy Pan-HER inhibitors Antibody drug conjugates: ado-trastuzumab emtansine, trastuzumab deruxtecan HER2 Exon 20 inhibitors: Mobocertinib, Poziotinib
ROS1 gene rearrangements	Different fusion partners	0.7–1.7%	ROS1, ALK and MET inhibitor: Crizotinib ROS1 and ALK inhibitors: Ceritinib, Lorlatinib ROS1, ALK and pan-TRK inhibitor: Entrectinib ROS1 and pan-TRK inhibitors: Repotrectinib, Taletrectinib
RET gene rearrangements	RET-KIF5B	1–2%	RET inhibitors: Selpercatinib, Pralsetinib
MET alterations	MET amplification Exon 14 skipping mutations	4–5% 2–4%	MET, ALK, and ROS1 inhibitor: Crizotinib MET inhibitors: Capmatinib, Tepotinib
NTRK gene fusions	NTRK 1, 2, 3 with different fusion partners	0.2%	Pan-TRK, ALK and ROS1 inhibitor: Entrectinib Pan-TRK inhibitor: Larotrectinib

# References to the frequencies of the genomic alterations can be found in the text. The frequencies vary considerably. The here reported numbers refer to a Caucasian population. KRAS: kirsten rat sarcoma viral oncogene homolog; EGFR: epidermal growth factor receptor; ALK: anaplastic lymphoma kinase; EML4: echinoderm microtubule-associated protein-like 4; BRAF: v-Raf murine sarcoma viral oncogene homolog B; HER2: human epidermal growth factor receptor 2; ROS1: c-ros oncogene 1; RET: rearranged during transfection; KIF5B: kinesin family member 5B gene; MET: c-MET; NTRK: neurotrophic tyrosine receptor kinase.

**Table 2:** Selected clinical trials of targeted agents.

Target	Agent	Trial	Phase	Treatment Line	N	Comparator Arm	ORR (%)	mPFS (Months)	mOS (Months)
KRAS G12C	*Sotorasib*	CodeBreaK100	I	Later-line	59	no	32%	6.3	NA
EGFR	*Erlotinib*	ENSURE	III	First-line	217	Cis/Gem	63% vs. 34%	11.0 vs. 5.6	26.3 vs. 25.5
		EURTAC	III	First-line	173	Platinum/Doce or Gem	64% vs. 18%	9.7 vs. 5.2	22.9 vs. 19.6
		OPTIMAL	III	First-line	154	Carbo/Gem	83% vs. 36%	13.1 vs. 4.6	22.8 vs. 27.2
	*Gefitinib*	IPASS	III	First-line	261/186 ^1^	Carbo/Pac	67% vs. 41% ^1^	10.9 vs. 7.4 ^1^	21.6 vs. 21.9 ^1^
		NEJ002	III	First-line	230 ^2^	Carbo/Pac	74% vs. 31% ^2^	10.8 vs. 5.4 ^2^	27.2 vs. 26.6 ^2^
		WJTOG 3405	III	First-line	177	Cis/Doce	62% vs. 32%	9.2 vs. 6.3	34.8 vs. 37.3
	*Afatinib*	LUX-Lung 3	III	First-line	345	Cis/Pem	56% vs. 23%	11.1 vs. 6.9	28.2 vs. 28.2
		LUX-Lung 7	IIB	First-line	319	Gefitinib	70% vs. 56%	11.0 vs. 10.9	27.9 vs. 24.5
	*Dacomitinib*	ARCHER 1050	III	First-line	452	Gefitinib	75% vs. 72%	14.7 vs. 9.2	34.1 vs. 27.0
	*Osimertinib*	FLAURA	III	First-line	556	Erlotinib or Gefitinib	80% vs. 76%	18.9 vs. 10.2	38.6 vs. 31.8
ALK	*Crizotinib*	PROFILE 1007	III	Later-line	347	Pem or Doce	65% vs. 20%	7.7 vs. 3.0	21.7 vs. 21.9
		PROFILE 1014	III	First-line	343	Platinum/Pem	74% vs. 45%	10.9 vs. 7.0	NR vs. 47.5
	*Ceritinib*	ASCEND-4	III	First-line	376	Platinum/Pem	73% vs. 27%	16.6 vs. 8.1	NR vs. 26.2
	*Alectinib*	ALEX	III	First-line	303	Crizotinib	83% vs. 76%	25.7 vs. 10.4	NR vs. 57.4
	*Brigatinib*	ALTA-1L	III	First-line ^3^	275	Crizotinib	74% vs. 62%	24.0 vs. 11.0	NR vs. NR
	*Ensartinib*	eXALT-3	III	First-line ^3^	290	Crizotinib	75% vs. 67%	25.8 vs. 12.7	Immature
	*Lorlatinib*	CROWN	III	First-line ^3^	296	Crizotinib	76% vs. 58%	NR vs. 9.3	Immature
BRAF	*Dabrafenib + Trametinib*	NCT01336634	II	First-line	36	No	64% ^4^	10.8 ^4^	17.3
				Later-line	57	No	68% ^4^	10.2 ^4^	18.2
HER2	*T-DM1*	NCT02675829	II	Different lines	18	No	44% ^4^	5.0 ^4^	NA
	*T-DXd*	DESTINY-Lung01	II	Later-line	42	No	62%	14	NA
ROS1	*Crizotinib*	PROFILE 1001	I	Different lines	53	No	72% ^4^	19.3 ^4^	51.4
	*Ceritinib*	NCT01964157	II	Later-line	32 (30 ^5^)	No	62% (67% ^5^)	9.3 (19.3 ^5^)	24
	*Lorlatinib*	NCT01970865	I–II	Different lines	69 (21 ^6^)	No	41% (62% ^6^)	NA (21.0 ^6^)	NA
	*Entrectinib*	STARTRK-1, STARTRK-2, ALKA-372-001	I–II	Different lines	53	No	77%	19	NR
RET	*Selpercatinib*	Libretto-001	I–II	Later-line/First-line	105/39	No	64%/85%	16.5/NR	NR/NR
	*Pralsetinib*	ARROW	I–II	Later-line/First-line	80/26	No	61%/73%	NA/NA	NA/NA
MET exon 14 skipping	*Crizotinib*	PROFILE 1001	I	Different lines	69	No	32% ^4, 7^	7.3 ^4^	20.5
	*Capmatinib*	GEOMETRY mono-1	II	Later-line/First-line	69/28	No	41%/68%	5.4/12.4	NA/NA
	*Tepotinib*	VISION	II	Different lines	152 (99 ^8^)	No	46% ^8^	8.5 ^8^	Immature
MET amplification	*Capmatinib*	GEOMETRY mono-1	II	Later-line/First-line	69/15	No	29%/40%	4.1/4.2	NA/NA
NTRK	*Entrectinib*	STARTRK-1,	I–II	Different lines	10	No	70%	NA	NA
		STARTRK-2,							
		ALKA-372-001							
	*Larotrectinib*	NCT02122913,	I–II	Different lines	12	No	75% ^4^	NA	NA
		NCT02637687,							
		NCT02576431							

References for all mentioned trials are found in the text. ORR: blinded independent review committee (BICR)-assessed overall response rate; mPFS: BICR-assessed median progression-free survival; mOS: median overall survival; NA: not available; NR; not reached. Cis/Gem: cisplatin and gemcitabine; Platinum/Doce or Gem: platinum (cisplatin or carboplatin) and either docetaxel or gemcitabine; Carbo/Gem: carboplatin and gemcitabine; Carbo/Pac: carboplatin and paclitaxel; Cis/Doce: cisplatin and docetaxel; Cis/Pem: cisplatin and pemetrexed; Pem or Doce: pemetrexed or docetaxel; Platinum/Pem: platinum (cisplatin or carboplatin) plus pemetrexed; T-DM1: ado-trastuzumab emtansine; T-DXd: trastuzumab deruxtecan. ^1^ The overall trial population included 1217 patients. 261 patients were positive for an EGFR mutation. As per protocol, outcome was assessed by local investigators. A post-hoc analysis by BICR was published later and included 186 patients with available CT scans. ORR and PFS is presented for these 186 patients, OS for the 261 patients. ^2^ The study assigned 230 patients to each treatment arm. OS and ORR were assessed for the 228 patients in the intention-to-treat (ITT) population. The PFS-population included 224 patients. ^3^ Prior chemotherapy allowed. ^4^ Investigator-assessed ORR and PFS. ^5^ Crizotinib-naïve patients. ^6^ TKI-naïve patients. ^7^ ORR was assessed in 65 patients (response-evaluable). ^8^ Efficacy parameters were only assessed for 99 patients.
